# Risk factors and foetal growth restriction associated with expectant treatment of early-onset preeclampsia

**DOI:** 10.1080/07853890.2022.2144642

**Published:** 2022-11-16

**Authors:** Jiao Yi, Lei Chen, Xianglian Meng, Yi Chen

**Affiliations:** Department of Obstetrics and Gynecology, Maternal and Child Health Care Hospital Affiliated with Anhui Medical University, Anhui Maternal and Child Health Care Hospital, Hefei, China

**Keywords:** Preeclampsia, early onset preeclampsia, foetal growth restriction, expectant treatment, 24-h proteinuria

## Abstract

**Objective:**

To identify the factors affecting expectant management of early-onset preeclampsia, and evaluate the correlation between expectant treatment and foetal growth restriction.

**Materials and Methods:**

The retrospective study included 72 women who were admitted for early-onset preeclampsia between February 2018 to April 2021. Data included maternal clinical parameters, demographic and maternal and neonatal outcomes, which were analysed for correlation.

**Results:**

Multiple logistic regression analysis demonstrated that the time interval from the onset of 24-h proteinuria to termination of pregnancy showed a strong correlation with the expectant treatment; Univariate logistic analysis confirmed that there was no correlation between expectant treatment and foetal growth restriction.

**Conclusion:**

There was a negative correlation between the duration of 24-h proteinuria and the expectant treatment of patients with early-onset preeclampsia; Expectant treatment could not improve the development of foetal growth restriction in patients with early-onset preeclampsia.

**KEY MESSAGES**
- The duration of 24-h proteinuria affects the effectiveness of expectant management of early-onset preeclampsia.
- Expectant management can reduce adverse neonatal outcomes due to iatrogenic preterm delivery, but it cannot improve the occurrence of foetal growth restriction.

## Introduction

Preeclampsia is a common and multi-system disorder of pregnancy characterized by the new onset of hypertension, proteinuria, or hypertension and end-organ dysfunction with or without proteinuria after 20 weeks of gestation [1]. The incidence of preeclampsia varies according to the particularities of the population studied in the literature. In developed countries, preeclampsia could complicate 1–10% of all pregnancies, while in developing countries a prevalence of 17% has been reported [2]. Women with preeclampsia are at increased risk for life-threatening obstetric or foetal complications, with 76,000 women and 500,000 babies dying each year [3], which is especially high in low-income countries.

Clinical manifestations of preeclampsia are diverse, and the pathogenesis is not fully understood. According to varied gestational ages and variations in severity, preeclampsia has been recognized as two different conditions: early onset preeclampsia (EP) occurs before 34 weeks of gestation, and late onset preeclampsia (LP) occurs at 34 weeks or later [4]. EP is a distinct and more severe clinical entity than LP [5], and is also less prevalent than LP, which accounts for only a small proportion of preeclampsia cases (<20%) [6]. From a pathophysiological point of view, EP and LP are considered two different conditions [7]. The main pathophysiology in EP is poor placentation secondary to defective remodelling of the uterine spiral arteries [8], in contrast, the LP is characterized by an increasing mismatch between normal maternal perfusion, coupled with a maternal predisposition to inflammation [9]. Thus, EP is associated with significant maternal and foetal morbidity and mortality. Research found that there were significant differences observed in the gestational age at delivery, foetal growth restriction (FGR) and birth weight in EP patients [10]. Women with EP had an increased chance of lower Apgar scores and longer neonatal hospitalization was also reported by others [11]. In a study including women with EP, the incidence of small for gestational age (SGA), delivery before 34 weeks, perinatal mortality, and caesarean section rates was high [12]. Conversely, LP is associated with minor placental involvement and milder clinical disease [13]. Study found that LP was less prone to complications and was associated with a well-grown offspring, especially had a higher rate of delivering large-for-gestational age infants [14].

In early-onset preeclampsia pregnancies, an iatrogenic preterm delivery is a common consequence as delivery is the definitive treatment and the safest option for the mother, which confronts clinicians with the challenge of preterm birth at very early gestational age. Study suggested that babies whose mothers were in the subgroup of expectant treatment had lower intraventricular haemorrhage, respiratory distress caused by hyaline membrane disease and higher gestation at birth [15]. Moreover, more advanced gestational age at delivery was associated with favourable neonatal outcome [16]. Therefore, the American College of Obstetricians and Gynaecologist (ACOG) Task Force on Hypertension in Pregnancy mentioned that EP without severe features may have benefits from expectant management of inpatient or outpatient [17]. Nevertheless, factors affecting expectant management have not been adequately studied, and as one of the most common complications of EP, there is no literature concerning whether expectant treatment can improve the development of FGR. Therefore, this retrospective study is conducted to estimate the factors affecting expectant treatment of EP patients, and investigate the influence of expectant treatment on FGR.

## Materials and methods

### Study design

This study was performed at a tertiary Obstetrics and Gynecology Center between February 2018 to April 2021. It is the largest tertiary obstetrics and gynaecology hospital in the region, with 20,000 deliveries on average per year, all the EP patients in the province are referred to this centre for further treatment. Only women with singleton pregnancy were enrolled, and women with pre-existing hypertension, previously diabetes mellitus, pre-gestational renal or hepatic disorders, thrombophilia, the presence of congenital anomalies and rheumatic immune disease were excluded. Because of the retrospective design of the study, the need to obtain informed consent from eligible women was waived, and this retrospective research was approved by the institutional ethics committee of the Anhui maternal and Child health care hospital.

Over the 3-year period, 72 pregnant women were diagnosed with EP. The expectant treatment was defined as expediting the delivery at least 24–48 h after corticosteroid administration [15].

In our hospital, for pregnant women in the presence of hypertension or oedema, a spot urine analyses is performed at every outpatient visit. Once the result expresses as +, the amount of protein excreted in the urine within 24 h will be measured. For inpatients with EP related symptoms and signs rather than proteinuria, 24-h total proteinuria will still be detected first. During hospitalization, in case of the maternal–foetal condition is stable, no matter what the level of proteinuria is, laboratory evaluation will be performed twice a week including 24-h proteinuria, in case of abnormalities, laboratory tests are repeated daily.

### Definitions

Preeclampsia is defined as a blood pressure reading of 140/90 mmHg or greater, measured on two separate times 4 h apart after 20 weeks of gestation, co-existing with proteinuria, or without proteinuria but accompanied by at least one of the following end-organ dysfunctions: renal involvement, impaired liver function, pulmonary oedema and prodromal signs like visual and cerebral symptoms [1].

Early onset preeclampsia is described as preeclampsia diagnosed and delivery before 34 weeks of gestation [4].

HELLP syndrome refers to a syndrome consisted of haemolysis, thrombocytopenia, and elevation of hepatic transaminases twice the upper limit of normality [18].

Proteinuria is described as more than 300 mg of protein in a 24-h collected urine sample [17].

Foetal intrauterine growth restriction (FGR) refers to an estimated weight on ultrasonographic examination below the 10th percentile adjusted to gestational age [19].

### Expectant treatment protocol

Relevant guidelines for the expectant treatment of EP have yet to be unified in China, clinical treatment of EP remains solely dependent upon the experience of the obstetrician. In our centre, all eligible women on the day of admission will be given oxygen, bed rest. If necessary, anti-hypertensive medication to stabilize blood pressure. Corticosteroids administered for foetus lung maturation and magnesium sulphate to prevent eclampsia are all given. During expectant management, maternal and foetal conditions are kept under surveillance, including daily monitoring of maternal weight, blood pressure, fluid intake and urine volume, as well as laboratory evaluation twice a week. In case of maternal condition is unstable, laboratory tests are repeated daily; Regard to foetal monitoring, foetal heart rate monitoring is performed at least once a day during admission, ultrasound to assess biophysical activity, amniotic fluid index, foetal growth, umbilical artery, middle cerebral artery is performed at a minimum frequency of twice weekly.

Indications for termination of pregnancy include: maternal organ dysfunction, pulmonary oedema, HELLP syndrome, suspected placental abruption, non-reassuring foetal status, or severe, uncontrolled hypertension, severe foetal growth restriction, and when 34 weeks’ gestation is reached.

### Data collection

Demographic characteristics, medical histories, and obstetric data on the pregnant women were collected, information regarding the course of pregnancy outcomes were also recorded, including: maternal age, gravidity, parity, body mass index (BMI), gestational week of delivery, history of hypertension in previous pregnancy, and anti-hypertensive treatment before hospitalization. Initial-onset symptoms (IOS) that appeared in patients later diagnosed with EP, gestational week of IOS onset, and the duration between IOS onset and delivery. Gestational weeks of hypertension, oedema and proteinuria onset respectively, and the interval from hypertension, oedema and proteinuria onset to delivery respectively. Initiation of anti-hypertensive treatment during hospitalization, indications for termination of pregnancy, and mode of delivery.

Maternal clinical and laboratory data were obtained from medical records, including: maximum systolic blood pressure (SBP), and maximum diastolic blood pressure (DBP). The maximum values of creatinine (Cr), uric acid (UA), 24-h proteinuria, alanine aminotransferase (ALT), aspartate aminotransferase (AST), and B-type natriuretic peptide (BNP) before delivery. The minimum values of total protein, albumin and platelet before delivery.

Neonatal outcomes referred to the occurrence of intrauterine demise, neonatal death, FGR and Apgar score.

All the data were from a clinical workstation.

### Statistical analysis

The statistical analyses were performed by SPSS 13.0 statistical packages (SPSS Inc., Chicago, USA.). Counting variables were expressed by percentage. Continuous variables were expressed by mean and standard deviation (SD). The multiple logistic regression analysis was for the correlation analysis between the expectant treatment and various parameters, and univariate logistic analysis was for the correlation analysis between the expectant treatment and foetal intrauterine growth restriction. Rank correlation analysis (Spearman correlation analysis) was performed to estimate the correlation between the expectant treatment and maternal and pregnancy parameters. A *p* < 0.05 was used for statistical significance.

## Results

From February 2018 until April 2021, 75 pregnant women were identified as EP. Three cases were excluded secondary to the complication of EP and the brevity between clinical presentation and delivery: one case experienced placental abruption and two cases were due to acute foetal distress, who were all underwent emergency caesarean section on the day of admission. Overall, 72 women were included for the final analysis.

Maternal characteristics, clinical information before expectant treatment are depicted in Table 1. The mean age of 72 EP women was 29.64 ± 5.23 years old (18–44 years), and with an average BMI of 25.13 ± 3.93 kg/m^2^ (18.6–37.59 kg/m^2^). The proportion of EP women with one gravidity, two gravidities and more than two gravidities were 29.2% (21/72), 37.5% (27/72), and 33.4% (24/72), respectively, and the percentage of primipara was 51.4% (37/72). Only 15.3% (11/72) EP patients experienced anti-hypertensive treatment before hospitalization. 15.3% (11/72) of patients reported hypertension history of a previous pregnancy and 5.6% (4/72) of patients suffered polycystic ovary syndrome before pregnancy. Mean gestational age at delivery was 31.58 ± 1.95 weeks (26.2–34 weeks). The indications for termination of pregnancy were: severe oedema with pleural effusion and ascites (4/72, 5.6%), foetal distress (23/72, 31.9%), uncontrolled hypertension (17/72, 23.6%), maternal organ dysfunction (18/72, 25%), FGR (4/72, 5.6%), and HELLP syndrome (6/72, 8.3%). The majority of patients underwent caesarean section (66/72, 91.7%), and the remaining 6 patients chose odinopoeia because of their serious condition. The mean expectant treatment was 8.15 ± 7.51 days (1–31 days). Initial-onset symptoms (IOS) of 72 EP women were oedema (35/72, 48.6%), hypertension (34/72, 47.2%) and FGR (3/72, 4.2%), respectively. The mean gestational weeks of the onset of IOS, hypertension, oedema and proteinuria were 28.05 ± 2.65 weeks (20–33.1 weeks), 29.05 ± 2.86 weeks (21.5–34 weeks), 28.73 ± 2.66 weeks (20–33.1 weeks), and 29.56 ± 2.74 weeks (21.2–34 weeks), respectively. The average time interval from the onset of IOS, hypertension, oedema and proteinuria to termination of pregnancy was 25.64 ± 15.6 days (3–83 days), 18.58 ± 15.96 days (1–72 days), 21.73 ± 14.26 days (1–83 days), and 14.93 ± 14.81 days (1–62 days), respectively.

**Table 1.** Maternal characteristics, clinical characteristics before expectant treatment.

Characteristics	$\bar{x}\pm s$ [*n*(%)]
Mean age (years)	29.64 ± 5.23
Mean BMI (kg/m²)	25.13 ± 3.93
Gravidity	
1	21(29.2%)
2	27(37.5%)
≥3	24(33.4%)
Parity	
0	37(51.4%)
1	31(43.1%)
2	4(5.6%)
Anti-hypertensive therapy before hospitalization	
No	61(84.7%)
Yes	11(15.3%)
Past history	
No	57(79.2%)
Hypertension	11(15.3%)
PCOS	4(5.6%)
Mean gestational age of delivery (weeks)	31.58 ± 1.95
Indications for termination of pregnancy	
Oedema	4(5.6%)
Foetal distress	23(31.9%)
Uncontrolled hypertension	17(23.6%)
Organ dysfunction	18(25%)
FGR	4(5.6%)
HELLP syndrome	6(8.3%)
Mode of delivery	
Odinopoeia	6(8.3%)
CS	66(91.7%)
Mean gestational age of IOS onset (weeks)	28.05 ± 2.65
Mean interval from IOS onset to delivery (days)	25.64 ± 15.6
IOS	
Oedema	35(48.6%)
Hypertension	34(47.2%)
FGR	3(4.2%)
Mean gestational age of hypertension onset (weeks)	29.05 ± 2.86
Mean interval from hypertension onset to delivery (days)	18.58 ± 15.96
Mean gestational age of oedema onset (weeks)	28.73 ± 2.66
Mean interval from oedema onset to delivery (days)	21.73 ± 14.26
Mean gestational age of proteinuria onset (weeks)	29.56 ± 2.74
Mean interval from proteinuria onset to delivery (days)	14.93 ± 14.81
Mean expectant treatment time (days)	8.15 ± 7.51

BMI: body mass index; PCOS: polycystic ovary syndrome; FGR: foetal growth restriction; CS: caesarean section; IOS: initial-onset symptoms.

Maternal blood pressure and laboratory parameters during expectant treatment are shown in Table 2. In the period of expectant treatment, the mean maximum SBP and DBP were 156.62 ± 18.34 mmHg (112–226 mmHg), and 103.89 ± 11.38 mmHg (65–130 mmHg), respectively. The mean maximum 24-h proteinuria was 4667.44 ± 2300.29 mg (119–13,806 mg). The mean maximum ALT and AST were 31.31 ± 31.71 IU/L (5.3–173.7 IU/L), and 33.17 ± 21.7 IU/L (12.7–112.5 IU/L), respectively. The mean maximum Cr and UA were 61.63 ± 16.45 µmol/L (32.4–102.5 µmol/L), and 474.66 ± 94.98 µmol/L (224.6–687.7 µmol/L), respectively. Mean maximum BNP was 926.49 ± 1509.46 pg/mL (100–9390 pg/mL). The mean minimum total protein and albumin were 50.14 ± 4.55 g/L (39.1–58.8 g/L), and 27.01 ± 3.39 g/L (18.7–33.4 g/L), respectively. The mean minimum platelet was 175.78 ± 110.33 × 10^9^/L (42–356 × 10^9^/L).

**Table 2.** Maternal blood pressure and laboratory parameters during expectant treatment.

Parameters	$\bar{x}\pm s$ [*n*(%)]
Mean SBP (mm/Hg)	156.62 ± 18.34
Mean DBP (mm/Hg)	103.89 ± 11.38
Mean Cr (µmol/L)	61.63 ± 16.45
Mean UA (µmol/L)	474.66 ± 94.98
Median of 24 h proteinuria (mg/24 h)	4667.44 ± 2300.29
Median of total protein (g/L)	50.14 ± 4.55
Median of albumin (g/L)	27.01 ± 3.39
Median of ALT (IU/L)	31.31 ± 31.71
Median of AST (IU/L)	33.17 ± 21.7
Median of platelet value (10^9^/L)	175.78 ± 110.33
Mean BNP (pg/mL)	926.49 ± 1509.46

SBP: systolic blood pressure; DBP: diastolic blood pressure; Cr: creatinine; UA: uric acid; ALT: alanine aminotransferase; AST: aspartate aminotransferase; BNP: B-type natriuretic peptide.

Correlation analysis between the expectant treatment of 72 EP women and various parameters is presented in Table 3. Statistically significant correlation was found between gestational weeks of the onset of IOS, hypertension, oedema and expectant treatment (*p* < 0.05). In addition, statistically significant association was also noted between the time interval from the onset of IOS, hypertension, oedema and 24-h proteinuria to termination of pregnancy and expectant treatment (*p* < 0.05). BNP also showed a strong correlation with expectant treatment (*p* < 0.05).

**Table 3.** Correlation analysis between the expectant treatment of 72 EP women and various parameters.

Parameters	*r*_s_	*p*
Maternal age (years)	−0.020	0.864
BMI (kg/m²)	0.033	0.782
Gravidity	0.029	0.357
Parity	0.081	0.055
Gestational age of delivery (weeks)	0.075	0.533
Gestational age of IOS onset (weeks)	−0.238	0.045
Interval from IOS onset to delivery (days)	0.349	0.003
Gestational age of hypertension onset (weeks)	−0.258	0.029
Interval from hypertension onset to delivery (days)	0.388	0.001
Gestational age of oedema onset (weeks)	−0.414	0.002
Interval from oedema onset to delivery (days)	0.581	0.000
Gestational age of proteinuria onset (weeks)	−0.216	0.071
Interval from proteinuria onset to delivery (days)	0.356	0.002
SBP (mm/Hg)	−0.098	0.413
DBP (mm/Hg)	−0.171	0.150
Cr (µmol/L)	−0.192	0.106
UA (µmol/L)	0.010	0.935
24 h proteinuria (mg/24 h)	0.002	0.986
Total protein (g/L)	0.148	0.215
Albumin (g/L)	0.200	0.092
ALT (IU/L)	0.010	0.937
AST (IU/L)	−0.116	0.330
Platelet value (10^9^/L)	0.097	0.417
BNP (pg/mL)	−0.278	0.018

EP: early onset preeclampsia; BMI: body mass index; IOS: initial-onset symptoms; SBP: systolic blood pressure; DBP: diastolic blood pressure; Cr: creatinine; UA: uric acid; ALT: alanine aminotransferase; AST: aspartate aminotransferase; BNP: B-type natriuretic peptide.

The results of the multiple logistic regression analysis of the correlation among the expectant treatment of 72 EP women and various parameters based on Table 3 are shown in Table 4. The correlation between the expectant treatment and various parameters observed in Table 3 was diminished and became non-significant, only the time interval from the onset of 24-h proteinuria to termination of pregnancy shown a strong correlation with the expectant treatment (*p* < 0.05).

**Table 4.** Multiple logistic regression analysis of the correlation among the expectant treatment of 72 EP women and various parameters based on Table 3.

Parameters	*β*	se	*t*	*p*	95% CI	
Gestational age of IOS onset (weeks)	6.11	13.33	0.46	0.649	−20.76	32.99
Interval from IOS onset to delivery (days)	0.50	1.90	0.27	0.792	−3.32	4.33
Gestational age of hypertension onset (weeks)	1.69	6.61	0.26	0.800	−11.65	15.02
Interval from hypertension onset to delivery (days)	0.62	0.94	0.66	0.512	−1.28	2.52
Gestational age of oedema onset (weeks)	−7.98	11.32	−0.71	0.485	−30.82	14.85
Interval from oedema onset to delivery (days)	−0.69	1.62	−0.43	0.671	−3.95	2.57
Interval from proteinuria onset to delivery (days)	0.11	0.05	2.19	0.034	0.01	0.22
BNP (pg/mL)	0.00	0.00	−0.62	0.542	0.00	0.00

EP: early onset preeclampsia; IOS: initial-onset symptoms; BNP: B-type natriuretic peptide.

Table 5 shows that univariate logistic analysis confirmed that the expectant treatment had no correlation with FGR (*p* > 0.05).

**Table 5.** Univariate logistic analysis for the correlation between the expectant treatment and foetal growth restriction.

Parameter	*β*	Se	Wald *χ*^2^	*p*	OR (95% CI)
Expectant treatment (days)	0.031	0.043	0.543	0.461	1.032 (0.949, 1.122)

Neonatal outcomes are shown in Table 6. No intrauterine demise was found in the cases. The incidence of FGR was 77.8% (56/72); There were seven (9.7%, 7/72) neonatal deaths, of which six cases were termination of pregnancy due to serious condition, the remaining one neonatal death was as a result of severe asphyxia with Apgar score (1 and 5 min) of 1–1. There were two cases of severe neonatal asphyxia (2.8%, 2/72), including one with Apgar score (1 and 5 min) of 1–1 and another with Apgar score (1 and 5 min) of 3–9. Five foetuses were born with mild asphyxia (6.9%, 5/72), and neonatal outcomes were favourable after treatment.

**Table 6.** Neonatal outcomes of the EP patients.

Neonatal outcome	
FGR (*n*, %)	56 (77.8%)
1 min APGAR score < 7 (*n*, %)	5 (6.9%)
1 min APGAR score < 4 (*n*, %)	2 (2.8%)
Neonatal death (*n*, %)	7 (9.7%)
Intrauterine demise (*n*, %)	0

EP: early onset preeclampsia; FGR: foetal growth restriction.

## Discussion

Delivery of the foetus and placenta remains to date the only treatment option for EP. However, EP poses a management dilemma, foetal prematurity and its consequences, such as acute respiratory syndrome, intraventricular haemorrhage, sepsis, bronchopulmonary dysplasia are what the foetus needs to face [20]. Neonatal outcome of infants born to mothers with EP is worse, and their mortality and neonatal complication rates are greater [21]. Expectant management in women with EP may reduce neonatal complications. Retrospective study found that close follow up and postponing delivery in stable and appropriate pregnant women with preeclampsia would be beneficial for neonates [22]. In the protocol of expectant treatment, the administration of corticosteroid medication for foetal lung maturation and magnesium sulphate (MgSO_4_) for prevention of seizures is started for conventional 48-h period, which have been demonstrated to improve neonatal outcomes in preterm birth earlier than 34 weeks [23]. In addition, the use of MgSO_4_ for a short time (up to 48 h) also plays a role in foetal neuroprotection in early preterm birth. Therefore, the longer the expectant treatment, the better the prognosis for the foetus. However, expectant treatment is usually accompanied with the progressive deterioration of the maternal condition, and sometimes even life-threatening. Thus, understanding factors affecting the expectant management of EP patients can assist in guidelines for treatment of EP patients.

To the best of our knowledge, this is the first study in the literature to detailed and systematic analysis of the factors affecting the periods of expectant treatment of EP patients. As saw in Table 4, our results find that the expectant treatment of EP is negatively associated with the duration from the occurrence of proteinuria to delivery, in other words, the longer the duration of proteinuria, the shorter the periods for expectant treatment. It is well established that the imbalance between soluble pro-angiogenic and anti-angiogenic substances of preeclampsia patients is responsible for the endothelial injury [24], then the presence of endothelial dysfunction leads to the development of proteinuria and kidney injury [25]. A relationship between serum total protein levels and proteinuria severity at preeclampsia diagnosis and delivery has been demonstrated [26], therefore, proteinuria is an indication of kidney injury and hypoproteinemia. We can speculate that along with the prolonged duration of proteinuria, the incidence of maternal pregnancy complications such as kidney injury and hypoproteinemia also increased, leading to early termination of pregnancy, which in turn shortens the periods of expectant treatment. Our findings are in accordance with previously published data, finding that proteinuria was associated with EP and premature delivery [27], and patients who had proteinuria-onset preeclampsia were more likely to give birth at an earlier gestational age [28]. In our study, another interesting finding is that there is no correlation between the amount of proteinuria and the periods of expectant treatment of EP. Our finding is supported by existing literature showing that preeclampsia severity cannot be determined by the level of proteinuria [29, 30], and protein amounts should not be used to determine clinical decisions [31].

With regarding to FGR, which occurs in 56 of 72 (77.8%) women with EP in our study population. As far as we know, the placenta is essential for the development of the foetus serves as an organ for nourishing and gas exchange, and constantly supports the growth of the foetus [32]. Therefore, when the utero-placental insufficiency occurs that interrupts nutrients supply to the foetus, creating conditions for FGR initiation. Previously reported data stated that after excluding foetal congenital or chromosomal anomalies, utero-placental insufficiency was one of the most common causes for FGR [33]. From a pathological point of view, when inadequate remodelling of maternal spiral arteries leads to a high-resistance low-flow system of utero-placental insufficiency, FGR occurs [34]. Thus, specific placental disorders such as EP is associated with FGR. More recent study observed that preeclampsia was associated with small for gestational age and that the association was stronger with early-onset disease [35]. In a prospective longitudinal study, FGR was observed in 22.7% of the patients with EP, and this ratio was statistically higher than LP group [5]. In contrast to the above-mentioned findings, we find a higher percentage of FGR in our cohort. This may be due to our hospital is the biggest tertiary obstetrics centre in the region, where all the most severe cases are referred, leading to a high incidence of FGR. In addition, based on univariate logistic analysis, we find that expectant treatment cannot improve the occurrence of FGR, this is supported by a 2013 study in the Cochrane Database of Systematic Reviews, researchers found that babies born to mothers in the interventionist groups (early delivery group) of EP were less likely to be small-for-gestational age [15]. Taken together, this could be explained by the fact that expectant treatment in an attempt to allow foetal maturation could place the foetus in a harmful utero-placental insufficiency environment, which in turn to adversely affect the growth of the foetus.

### Strengths and limitations

The results of the present study may be limited by its retrospective single-centre design. However, as the largest tertiary obstetric centre in the region, the sample we studied is representative of the regional population; Because of the single-centre setting, we can obtain detailed patient information and laboratory parameters from the electronic system; In addition, diagnosis of proteinuria is performed according to the gold standard 24-h urine protein collections, as opposed to the insensitive dipstick measurement; Lastly, all the laboratory tests are tested in the same laboratory, from which test bias can be eliminated.

## Conclusions

In conclusion, this study shows that the duration of proteinuria is closely related to the expectant treatment of EP patients. Expectant treatment may be beneficial for neonates [22], however, it is not beneficial to improve the development of FGR. Further prospective studies with enlarged samples should be performed to confirm our conclusion, and to provide evidence-based base for expectant treatment of EP patients.

## Data Availability

The datasets analysed during the current study are available from the corresponding author on reasonable request.
